# Anti-Hyperalgesic Effect of Isopulegol Involves GABA and NMDA Receptors in a Paclitaxel-Induced Neuropathic Pain Model

**DOI:** 10.3390/ph18020256

**Published:** 2025-02-14

**Authors:** Deyna Martins, Boris Acha, Mickael Cavalcante, Suellen Pereira, Ana Viana, Flaviano Ribeiro Pinheiro-Neto, Priscyla Mendes, Dalton Dittz-Júnior, Francisco Oliveira, Tatiana Ventura, Maria da Graça Lobo, Fátima Ferreirinha, Paulo Correia-de-Sá, Fernanda Almeida

**Affiliations:** 1 Post Graduate Program in Pharmacology, Laboratory of Pain Pharmacology, Medicinal Plants Research Center, Federal University of Piauí—UFPI, Teresina 64049-550, Brazil; z.suellen@gmail.com (S.P.); flavianopinheiro993@gmail.com (F.R.P.-N.); priscylamendes04@gmail.com (P.M.); fassisol@ufpi.edu.br (F.O.); 2 Post Graduate Program in Biotechnology—RENORBIO, Federal University of Piauí—UFPI, Teresina 64049-550, Brazil; timah.boris@yahoo.com; 3 Laboratory of Experimental Cancerology, Medicinal Plants Research Center, Federal University of Piauí—UFPI, Teresina 64049-550, Brazil; mickael.laudrup14@gmail.com (M.C.); daltondittz@ufpi.edu.br (D.D.-J.); 4 Nursing Department, State University of Maranhão (UEMA)-Campus Santa Inês, Maranhão 65306-219, Brazil; flavia_seraine@hotmail.com; 5 Laboratório de Farmacologia e Neurobiologia, Centro de Investigação Farmacológica e Inovação Medicamentosa (MedInUP/RISE-Health), Instituto de Ciências Biomédicas de Abel Salazar Universidade do Porto (ICBAS-UP), 4050-313 Porto, Portugal; tatianaventura28@msn.com (T.V.); mglobo@icbas.up.pt (M.d.G.L.); mfferreirinha@icbas.up.pt (F.F.); farmacol@icbas.up.pt (P.C.-d.-S.)

**Keywords:** isopulegol, paclitaxel, von Frey, neuropathy, behavioral evaluation, pharmacological synergism, hyperalgesia

## Abstract

**Background**: Neuropathic pain can be triggered by chemotherapy drugs such as paclitaxel (PTX). Management of pain is limited by drugs’ ineffectiveness and adverse effects. Isopulegol (ISO) is a monoterpene present in the essential oils of several aromatic plants and has promising pharmacological activities. **Objectives**: to evaluate the antinociceptive activity of ISO in a PTX-induced neuropathic pain model. **Methods**: the toxicity of ISO was evaluated in healthy and cancerous cells. Behavioral assessments were performed using the von Frey and acetone tests. We investigated the involvement of the GABAergic pathway, NMDA, TNF-α, and the release of GABA and glutamate in the presence of ISO. **Results**: ISO showed little or no cytotoxicity in U87 and MDA-MB-231 cells. In both acute and subacute treatment, ISO at doses of 25, 50, and 100 mg/kg (* *p* < 0.05) increased the mechanical nociceptive threshold of neuropathic animals compared to the control group and reduced thermal sensitivity. Its action was reversed by pre-treatment with flumazenil and potentiated by the NMDA antagonist, MK-801. TNF-α and glutamate levels were reduced and GABA release was increased in the tests carried out. **Conclusions**: ISO shows low toxicity in neuronal cells and its association with PTX generated synergism in its cytotoxic action. The antinociceptive effect of ISO is due to activation of GABA and antagonism of NMDA receptors and involves the stabilization of neuronal plasma membranes leading to an imbalance in the release of neurotransmitters, favoring GABA-mediated inhibition over glutamatergic excitation.

## 1. Introduction

Neuropathic or neurogenic pain is caused by a lesion or disease of the somatosensory nervous system [1]. This type of pain has complex mechanisms with peripheral and central characteristics [2,3]. Recently, the number of cases of diseases that lead to the development of neuropathic pain has grown alarmingly. These include cancer, which can cause neuropathic pain in three ways: by compression and consequent damage to nerve endings, generated by the disorderly growth of tumors; by pharmacological treatment; or even by immunomediation, i.e., neurological disorders associated with the action of antibodies against neuronal antigens expressed by the tumor, known as paraneoplastic neuropathy [1,4,5,6,7].

Cancer chemotherapy is based on the use of antineoplastics derived from platinum, taxanes (paclitaxel), vinca alkaloids, thalidomide, and bortezomib, among others [8,9]. However, chemotherapy can also lead to harmful unintended consequences that can present themselves during treatment and persist as long-term sequelae in many cancer survivors, such as cancer-related fatigue (CRF), chemotherapy-induced peripheral neuropathy (CIPN), chemotherapy-related cognitive impairment (CRCI), and ovarian failure or infertility [10,11].

Studies have shown that among patients treated with paclitaxel (PTX), 80% reported symptoms of chemotherapy-induced peripheral neuropathy (CIPN) within 24 to 72 h after administration. It is estimated that 25% of these patients will require a reduction in the dose of paclitaxel [12,13]. This occurs in almost 98% of patients and is exacerbated when the cumulative dose exceeds 1400 mg/m^2^ [13]. Half of patients recover from CIPN caused by paclitaxel within 9 months. However, on average, 41% of patients exhibit prolonged negative effects from CIPN lasting up to 3 years after treatment [14,15].

The treatment of neuropathic pain is challenging due to its heterogeneous etiologies, lack of objective diagnostic tools, and resistance to classic analgesic drugs [15]. In this context, several natural substances have been studied for their high therapeutic potential, stimulating the scientific community to carry out studies and pharmacological trials to investigate the possible action of these substances [16,17,18]. Isopulegol (ISO) is a monocyclic alcohol monoterpene present in the essential oils of various plants, such as *Corymbia citriodora* H., *Zanthoxylum schinifolium* L., and *Melissa officinalis* L. [19,20,21,22,23]. Paik et al. [20] showed that the essential oil of *Z. schinifolium* L., containing mostly ISO, is capable of inducing apoptosis in human hepatoma HepG2 cells, which suggests a plausible use of this oil as an anti-tumor agent in the therapy of hepatocellular carcinoma. ISO has antioxidant and anxiolytic [24], anticonvulsant and sedative [25], gastroprotective [26], and anti-inflammatory effects [27].

Studies carried out in our laboratory have shown that ISO has antinociceptive activity in acute pain through its action on the opioid system via K_ATP_ channels, participation of muscarinic receptors, and inhibition of nitric oxide and cGMP [28]. Knowing the pharmacological potential of monoterpenes and the promising activities demonstrated by ISO in antinociception in acute pain models, the present proposal aims to investigate the antinociceptive potential of ISO in paclitaxel-induced neuropathic pain in mice.

## 2. Results

### 2.1. Evaluation of ISO Cytotoxicity in Vitro

#### 2.1.1. Hypotonic Hemolysis with Rat Erythrocytes

**Figure 1:**
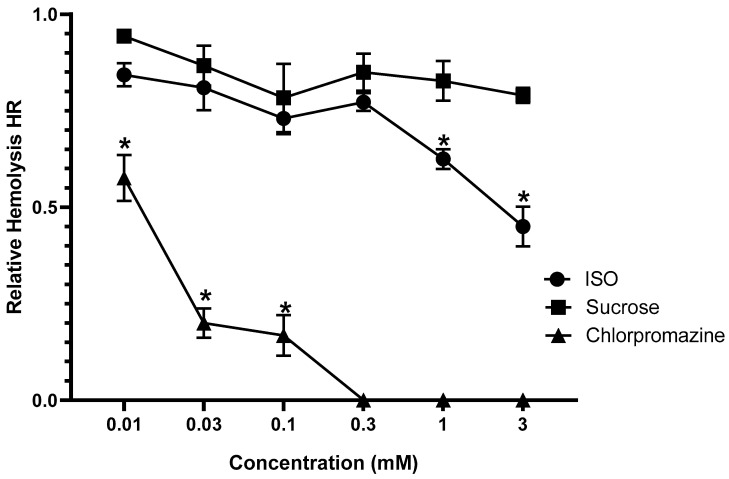
Action of ISO on the hypotonic-induced hemolysis of rat erythrocytes in vitro. Each group represents mean ± SEM of an *n* number of experiments (* *p* < 0.05 compared to the control group; two-way ANOVA).

At concentrations of 1 and 3 mM, ISO stabilized rat erythrocyte membranes and attenuated hemolysis when compared to the control, but this did not happen at concentrations lower than 0.3 mM. When used as a positive control, the antidepressant chlorpromazine (0.01, 0.03, and 0.1 mM) attenuated the hemolysis of rat erythrocytes (Figure 1).

#### 2.1.2. Effect of ISO on the Viability of U87 Glioblastoma and MDA-MB-231 Human Breast Carcinoma Cells in Vitro

**Figure 2:**
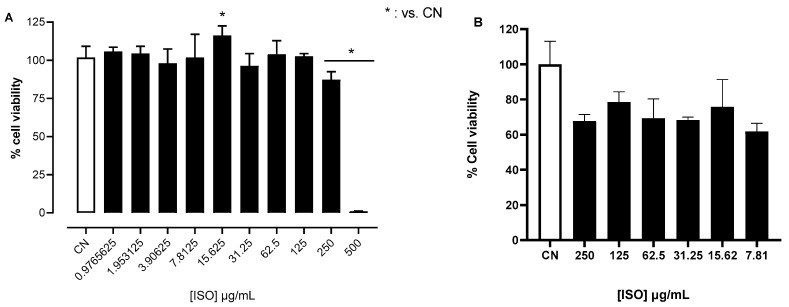
Effect of ISO on the viability of glioblastoma cells (U87) (**A**) and MDA-MB-23 cells (**B**). Each group represents the mean ± SEM (* *p* < 0.05 compared to control group; one-way ANOVA). CN = control. Cell viability was determined by the enzymatic conversion of resazurin to resorufin (readings at 570 and 600 nm).

In U87 glioblastoma cells, ISO presented cell toxicity at concentrations higher than 250 μg/mL (Figure 2A). ISO did not affect cell viability of MDA-MB-231 triple-negative breast carcinoma cells at the concentrations studied (Figure 2B).

#### 2.1.3. Evaluation of the Effect of ISO Associated with PTX on the Viability of MDA-MB-231 Human Breast Carcinoma Cells

**Figure 3:**
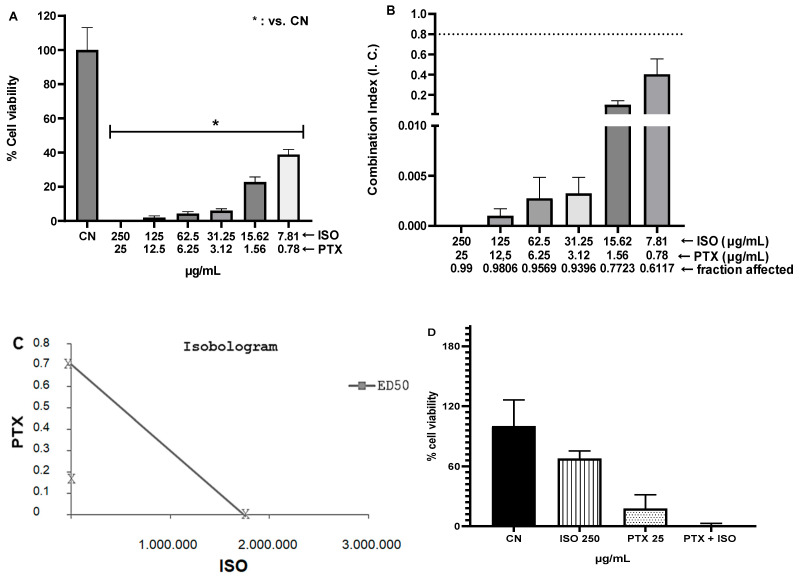
(**A**) Pharmacological synergism between ISO and PTX cytotoxicity on MDA-MB-231 Human Breast Carcinoma cells. (**B**,**C**) Combination index (CI) and fraction of affected cells (Fa) resulting from the fixed association of ISO and PTX at 1:10 ratio. CI > 1.2 indicates antagonism, CI < 0.8 indicates synergism, while CI = 0.8–1.2 indicates addition. In B the dashed line indicates the cut-off point for synergism (from 0.8 downwards). From this point we have addition, up to 1.0. Above 1.0 configures antagonism (**C**) Isobologram with IC50. The straight line indicates the IC50 of PTX on the y-axis and ISO on the x-axis. The x below the line indicates synergism of the ISO+PTX combination. (**D**) Percentage inhibition of cell viability of ISO + PTX. The results are expressed as the mean ± standard error of the mean of three independent experiments.

We used a fixed 1:10 association between PTX and ISO to determine the combination index (CI) and the fraction of affected cells (Fa) in the MDA-MB-23 Human Breast Carcinoma cell lineage. ISO played a concentration-dependent synergistic effect (CI < 0.8) with the cytotoxic action of PTX, yielding greater inhibition of cells viability (Figure 3).

### 2.2. Determination of Nociception by Mechanical Sensitivity and Cold Sensitivity

#### 2.2.1. Evaluation of the Acute Antinociceptive Action of ISO After Induction of Neuropathy by PTX

**Figure 4:**
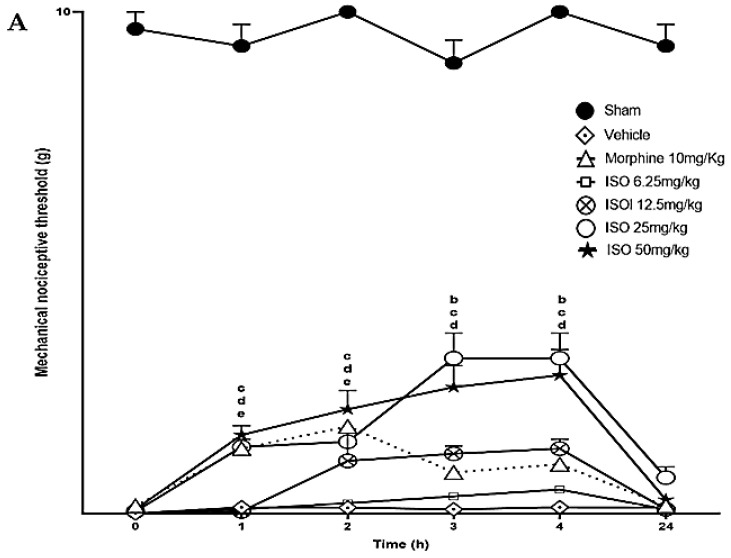

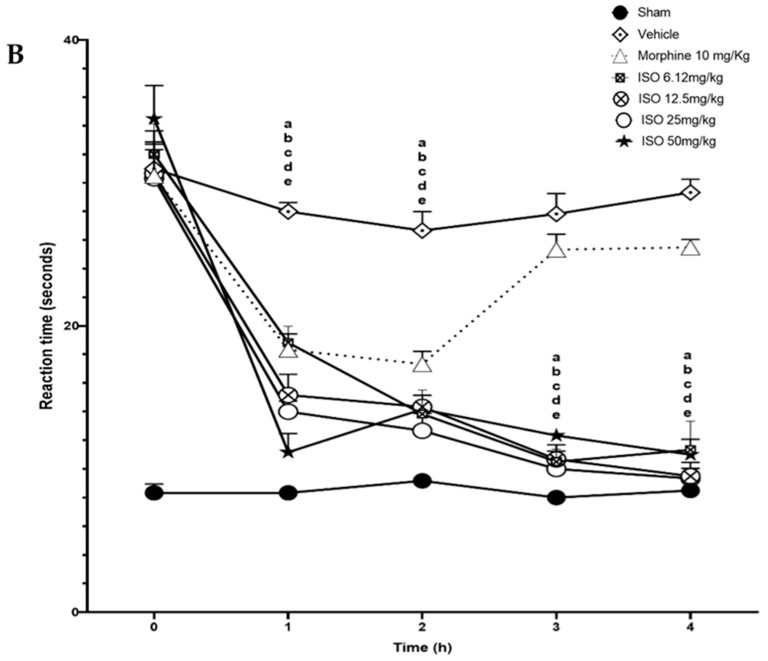
Mechanical and thermal nociceptive threshold of acute treatment with ISO in rats displaying sustained PTX-induced neuropathy. The animals (n = 6–8) were treated with a single dose of ISO (6.25, 12.5, 25, and 50 mg/kg, v.o.) on the 9th day of the protocol. The nociceptive threshold to mechanical stimuli was assessed by the von Frey test (**A**), and thermal sensitivity to cold stimuli was assessed by the acetone test (**B**), both on day 8 (baseline, defined as time 0) and on day 9, after 60, 120, 180, and 240 min and 24 h in the von Frey test. The significance levels of the groups were considered when *p* < 0.5 (the letters a, b, c, d, and e correspond to significance, referring to ISO at 6.25, 12.5, 25, and 50 mg/kg and morphine at 10 mg/kg, respectively) compared to the negative control (two-way ANOVA, post hoc Tukey test).

After confirming PTX-induced neuropathy establishment and maintenance, the animals were treated with a single dose of ISO (6.25, 12.5, 25, and 50 mg/kg, v.o.) or morphine (10 mg/kg, s.c.), and observed over a period of 4 h and 24 h (Figure 4A). At doses of 25 and 50 mg/kg, ISO significantly increased the mechanical nociceptive threshold of the animals (from 0.16 to 1.4–4 g), as assessed by the von Frey test at the 1 h time point; this analgesic effect lasted until the 4-h time point when compared to the negative control group (* *p* < 0.05). The anti-nociceptive action of ISO (12.5 mg/kg) delayed to be observed only at 3rd and 4th hour after application compared to the control group (* *p* < 0.05), while a lower dose (6.25 mg/kg) had no anti-nociceptive effect. Morphine (10 mg/kg), used as a positive control, significantly increased the animals’ mechanical nociceptive threshold in the first two hours, compared to the negative control group (* *p* < 0.5). There was no significant change in the nociceptive threshold of the animals in the control group. Evaluation of the mechanical pain threshold 24 h after application had no significant differences between PTX and control groups.

ISO decreased PTX-induced neuropathic cold sensitivity (revealed by the acetone test) 9 days after its administration (Figure 4B). Treatment with ISO (6.25, 12.5, 25, and 50 mg/kg, v.o.) significantly delayed (* *p* < 0.05) the reaction time of the animals to acetone at all times evaluated, compared to the negative control. For example, at a dose of 25 mg/kg, the reaction time decreased from ±30 s to ±12.6 s in the second hour, compared to the negative control. Morphine at 10 mg/kg transiently reduced the PTX-induced neuropathic cold sensitivity only from 60 to 120 min compared to the control group (* *p* < 0.5).

#### 2.2.2. Evaluation of the Subacute Antinociceptive Activity of ISO After Induction of Neuropathy by PTX

**Figure 5:**
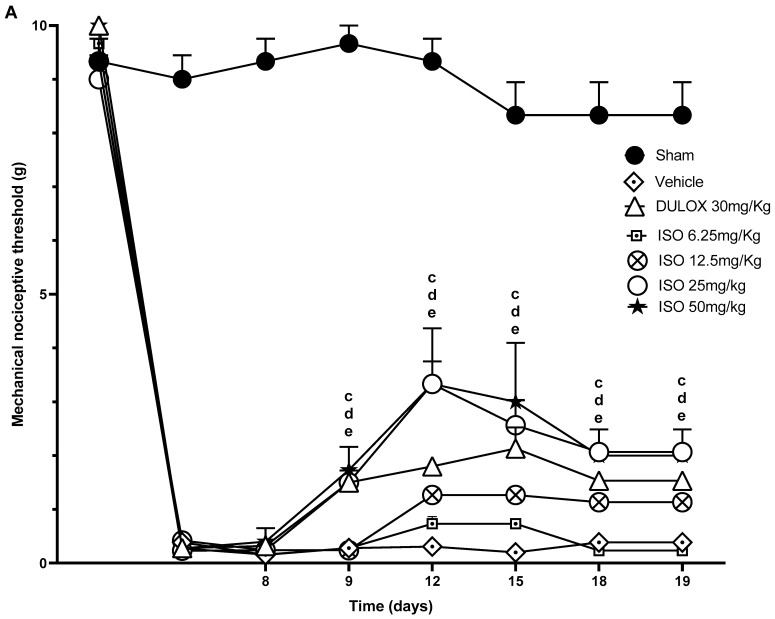

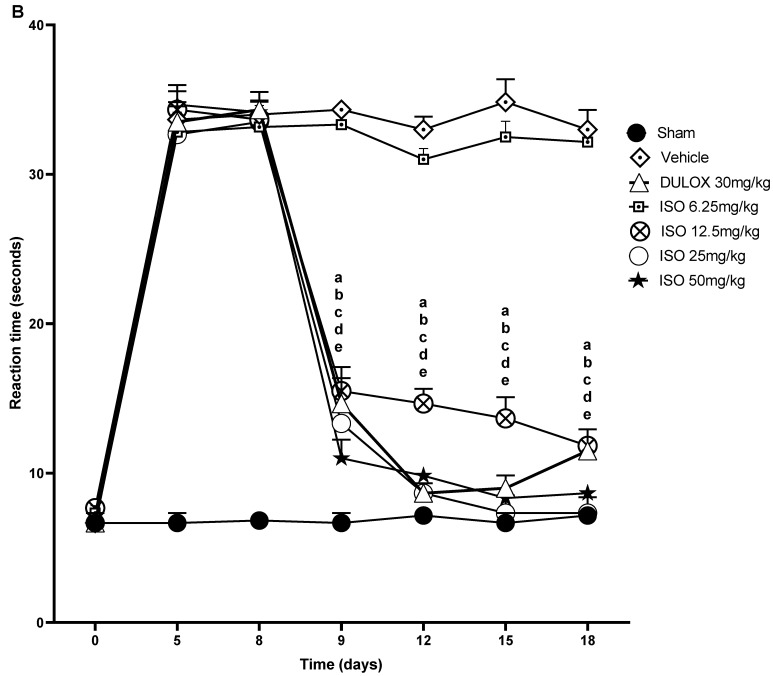
Mechanical and thermal nociceptive threshold of subacute treatment with ISO in rats displaying sustained PTX-induced neuropathy. The animals (n = 6–8) received daily treatment with ISO (6.25, 12.5, 25, and 50 mg/kg, v.o.) from the 9th to the 19th day of the protocol. The nociceptive threshold to mechanical stimulation was assessed using the von Frey test (**A**), and the thermal nociceptive threshold to cold was assessed using the acetone test (**B**) on days 9, 12, 15, and 18. The significance levels of the groups were considered significant when *p* < 0.05 (the letters a, b, c, d, and e correspond to significance for ISO at 6.25, 12.5, 25, and 50 mg/kg and duloxetine at 30 mg/kg, respectively) (two-way ANOVA, post hoc Tukey test).

Subacute treatment with ISO (6.25, 12.5, 25, and 50 mg/kg, v.o.) or duloxetine (30 mg/kg) was performed daily for 11 days (from day 9 to day 19 of the protocol). The mechanical and cold sensitivity tests were applied every two days. ISO (25 and 50 mg/kg) increased the mechanical nociceptive threshold of treated animals from day 9 until day 19 (* *p* < 0.05) compared to the negative control group; no significant differences were observed among increasing doses of ISO. Treatment with ISO (6.25 and 12.5 mg/kg) showed no effects on the nociceptive threshold of the animals compared to the negative control. Duloxetine (30 mg/kg) significantly increased the animals’ mechanical nociceptive threshold on all evaluation days (* *p* < 0.05) when compared to the control. On the 12th day of evaluation, ISO (25 mg/kg) was significantly more effective than duloxetine (30 mg/kg), thus increasing animals’ mechanical nociceptive threshold by more than 40% compared to the positive control (* *p* < 0.05) (Figure 5A).

ISO (12.5, 25, and 50 mg/kg) significantly reduced the neuropathic cold sensitivity induced by PTX from day 9 to day 19, when compared to the negative control group. The anti-nociceptive effect of ISO was dose dependent as the effect of the 12.5 mg/kg dose was inferior to 25, 50, and 100 mg/kg doses. At 25 mg/kg, ISO reduced the reaction time to acetone from 39 s to 12 s starting from the first day of treatment, but further increases in dosage failed to produce stronger effects. The 6.25 mg/kg dose was insufficient to produce analgesia under the present conditions. Duloxetine (30 mg/kg) reduced (*** *p* < 0.0001) the cold sensitivity score from day 9 to day 18, when compared to the negative control group (Figure 5B).

#### 2.2.3. Involvement of the GABA-A Receptor in the Antinociceptive Effect of ISO

**Figure 6:**
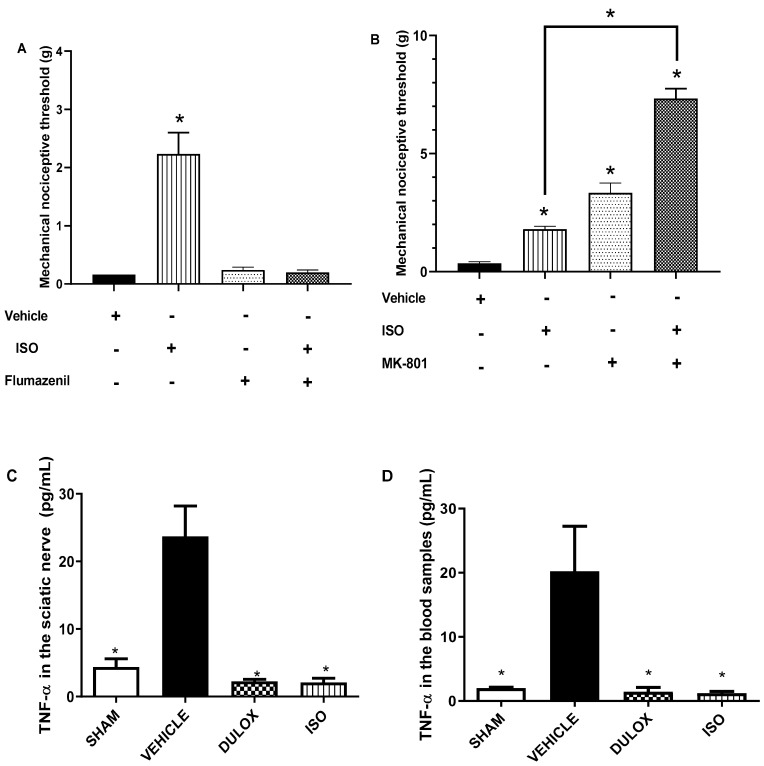
Involvement of GABA-A (**A**) and NMDA (**B**) receptors in the anti-nociceptive effect of ISO and quantification of TNF-αin pg/mL in the sciatic nerve (**C**) and blood samples (**D**) of animals (n = 6) with paclitaxel-induced neuropathy. Treatments involved ISO (25 mg/kg, v.o.), flumazenil (5 mg/kg, i.p.; a competitive inhibitor at the benzodiazepine recognition site of the GABA-A receptor), and MK-801 (0.03 mg/kg, i.p.; an NMDA receptor antagonist) applied alone or in combination; negative control consisted in the application of 2% Tween 80 and 0.9% NaCl. (+) indicates presence and (−) absence of treatment. The mechanical nociceptive threshold was determined after 1-h application using the von Frey test. * *p* < 0.05 compared to the negative control (one-way ANOVA, post hoc Tukey test). B * of the line indicates that there was a significant difference between the ISO and MK-801 groups.

Pre-treatment of the animals with flumazenil (5 mg/kg, i.p.), a competitive inhibitor at the benzodiazepine recognition site of the GABA-A receptor, significantly reversed the anti-nociceptive effect of ISO (25 mg/kg, v.o; * *p* < 0.05) on mechanical pain sensation in rats with PTX-induced neuropathy compared to the ones treated with ISO (25 mg/kg) alone (Figure 6A).

#### 2.2.4. Involvement of the NMDA Receptor in the Antinociceptive Effect of ISO

Pre-treatment of the animals with the glutamate NMDA receptor antagonist, MK-801 (3 mg/kg, i.p.), significantly reduced the mechanical pain sensation in rats with PTX-induced neuropathy. MK-801 (3 mg/kg, i.p.) had a synergistic anti-nociceptive effect to ISO (25 mg/kg, v.o.; * *p* < 0.05) when both drugs were applied to rats with PTX-induced neuropathic pain compared to the control group treated with ISO (25 mg/kg, v.o.) alone (Figure 6B).

#### 2.2.5. Quantification of TNF-α in the Sciatic Nerve of Neuropathic Animals Treated with ISO

The concentration of TNF-α was quantified in sciatic nerve and blood samples (Figure 6C and Figure 6D, respectively). TNF-α levels were assessed after subacute treatment with ISO (see above). Data show a 91.1% reduction (* *p* < 0.05) in the concentration of TNF-α when ISO was applied at the 25 mg/kg dose. The magnitude of this inhibitory effect was similar to that observed in the group treated with duloxetine (30 mg/kg), which caused a reduction of 90.3% in the concentration of TNF-α in the sciatic nerve compared to the negative control group. The blood concentration of TNF-α was also reduced (* *p* < 0.05) by 94% and 93% in animals treated with ISO (25 mg/kg) and duloxetine (30 mg/kg), respectively (Figure 6C), compared to the control group.

### 2.3. Effect of ISO on the Release of [^3^H]GABA and [^14^C]Glutamate from Symaptosomes of the Spinal Cord Depolarized by KCl

**Figure 7:**
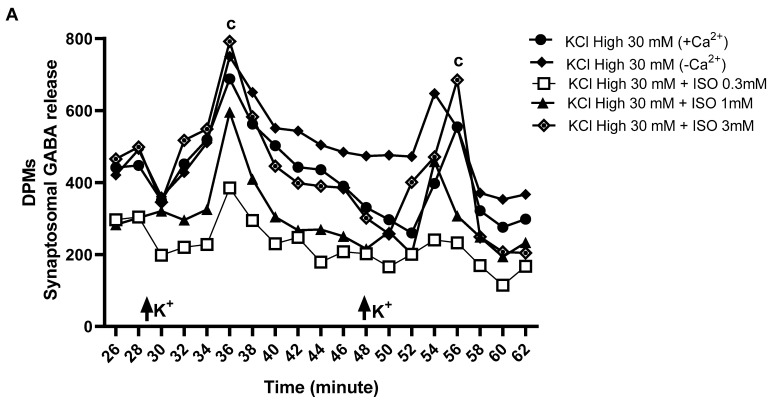

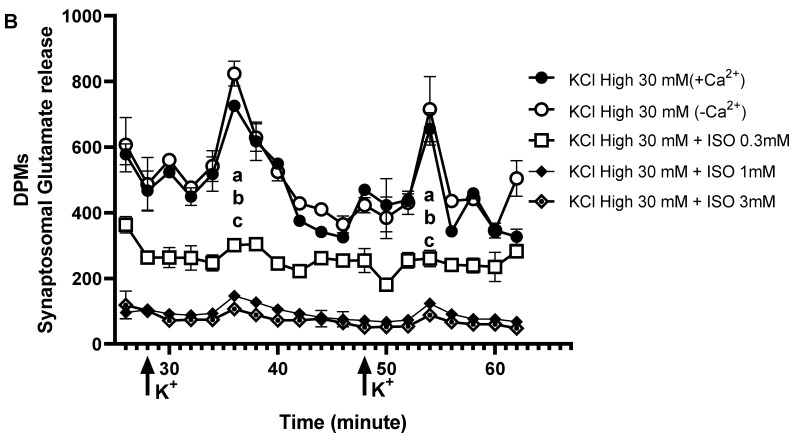
Effect of ISO on the release of [^3^H]GABA (**A**) and [^14^C]Glutamate (**B**) from symaptosomes of the rat spinal cord depolarized by KCl (30 mM). The radioactive content of the fractions collected and the remainder on the filters at the end of the protocol were assessed by liquid scintillation spectrometry. Each group represents the mean ± S.E.M of four animals. *p* < 0.05 compared to the control group (the letters a, b, c, correspond to significance for KCl High at 30 mM + ISO at 0.3 mM, KCl High at 30 mM + ISO at 1 mM, and KCl High at 30 mM + ISO at 3 mM, respectively) (two-way ANOVA, Bonferroni test).

ISO (3 mM) promoted the release of [^3^H]GABA from rat spinal cord synaptosomes depolarized with KCl (30 mM) by about 19.4% compared to the control group without any added drug (Figure 7A). Moreover, ISO applied at 0.3, 1.0, and 3.0 mM concentrations inhibited the release of [^14^C]Glutamate (* *p* < 0.05) from rat spinal cord synaptosomes depolarized with KCl (30 mM) by 59.7%, 80.4%, and 86%, respectively, compared to the control situation without addition of the drug (Figure 7B).

## 3. Discussion

Cumulative doses and the duration of PTX infusion are related to the severity of neurotoxicity [13]. Although various mechanisms underlying PTX-induced neurotoxicity have been advanced in the literature, there is still no effective treatment available in clinical practice to prevent or manage this clinical condition. Monoterpenes, which are found in the essential oils of aromatic plants, have emerged as potential alternatives for treating acute and chronic pain. Carvacrol, limonene, α-phellandrene, terpineol, linalyl acetate, and linalool are among the monoterpenes demonstrating anti-nociceptive activity, together with many other constituents of this superfamily showing promising effects [29,30,31,32].

ISO is a monocyclic alcohol monoterpene present in the essential oils of various aromatic plants and has shown promising antioxidant, anxiolytic [24], anticonvulsant, and sedative effects [25], in addition to gastroprotective [26] and acute antinociceptive activities [28]. Regarding toxicity, ISO exhibits low acute toxicity, with an oral LD_50_ in rats of 1.03 ± 0.10 g/kg and a cutaneous LD_50_ in rabbits of 3 g/kg [33]. Studies have also shown that acute oral treatment with ISO and ISO/β-CD using a single oral dose of 2000 or 5000 mg/kg did not produce any clinical signs of toxicity or death in the animals within 14 days, demonstrating low oral toxicity [27]. In tests with erythrocytes, ISO demonstrated the ability to partially protect against hypotonic hemolysis in rat erythrocytes, compared to sucrose (negative control) and chlorpromazine (with a protective effect, used as a positive control). The erythrocyte osmotic fragility test is a simple and sensitive test for evaluating drug-induced membrane stabilization at concentrations similar to those found in human plasma in vivo [34].

In addition, ISO was not very cytotoxic in U87 cells, implying that the ISO’s antinociceptive effect is not related to neuronal cell death but rather to other proposed mechanisms and pathways. However, although the major components of essential oils often reflect their biological activities, many researchers suggest that the bioactivity of an essential oil is rarely attributable to a single active compound. Instead, it is typically due to the synergistic action of various chemical substances, which may not necessarily be the most abundant [35,36]. This was observed in the effect of combining ISO with PTX on MDA-MB231 cells. In this study, ISO exhibited a synergistic inhibitory effect on the concentration-dependent cytotoxic action of PTX, leading to enhanced inhibition of cell viability.

Previous studies have shown that the action of ISO, at the doses investigated in this study, does not affect the animals’ motor coordination or exploratory behavior [25]. These findings suggest that ISO’s effects are likely mediated through inhibitory nociceptive pathways. In this study, intraperitoneal administration of PTX at 2 mg/kg for four consecutive days, resulting in a cumulative dose of 8 mg/kg, induced painful peripheral neuropathy in mice. Acute treatment with ISO significantly increased the animals’ mechanical nociceptive threshold. ISO at lower doses showed displayed delayed antinociceptive effect. This finding at lower ISO doses may be associated with its effect on inflammatory mediators involved in neuropathic pain, while at higher doses it can more powerfully alter other pain modulation pathways [27]. The antinociceptive action of ISO was more prolonged and superior to that of morphine, as this opioid only reduced the nociceptive threshold during the first two hours of evaluation, while ISO maintained a high threshold to mechanical and thermal sensation until the fourth hour. After 24 h, ISO showed no reduction in the mechanical pain threshold. Subacute treatment with ISO at the highest doses studied (mg/kg) was able to increase the mechanical nociceptive threshold of treated animals compared to negative controls, and this effect persisted throughout the treatment period (10 days).

Regarding cold sensitivity, treatment with ISO significantly attenuated the animals’ reaction time to acetone at the evaluated time points. In the subacute treatment, ISO significantly reduced the cold sensitivity score from the 9th to the 19th day compared to the negative control group.

Based on these data, we suggest that ISO exhibits acute and subacute antinociceptive activity in animals with neuropathic pain induced by PTX. Supporting these results, Gouveia and colleagues [30] demonstrated that α-terpineol, a monocyclic monoterpene with a molecular structure similar to that of ISO, exhibits an antinociceptive effect by increasing the mechanical nociceptive threshold in animals with PTX-induced neuropathic pain. Given ISO’s antiallodynic activity and its role as an intermediary in the production of menthol [37], these findings complement the studies by Brid and colleagues [38], which affirmed that 5% topical menthol is a potential analgesic for peripheral neuropathic pain induced by chemotherapy and post-mastectomy pain syndrome in humans.

It is also known, that neuropathic conditions cause reductions in the GABAergic inhibitory function [39]. The reduction in GABAergic inhibition caused by neuropathy is mediated by a decrease in GABA receptors expression. This is shown in a study by Li et al. [39], which demonstrated reduced expression of the δ subunit of the GABA-A receptor in neurons of the substantia gelatinosa (SG) of the dorsal horn of the spinal cord of neuropathic mice after sciatic nerve constriction.

Another study showed that treatment with bicuculline (a GABA-A receptor antagonist) or baclofen (a GABA-B receptor agonist) induced thermal hypersensitivity in healthy rats [40]. Conformational changes in these receptors, combined with the antinociceptive effects of GABA agonists, support the hypothesis that GABA receptors are involved in the onset and maintenance of neuropathic hyperalgesia [39,41]. Therefore, we evaluated animals pre-treated with flumazenil (5 mg/kg, i.p.), a competitive inhibitor at the benzodiazepine recognition site of the GABA-A receptor, and found that it significantly reversed the antinociceptive effect of ISO in animals with PTX-induced neuropathic pain. Studies have also shown that menthol not only increased currents induced by low concentrations of GABA but also directly activated the GABAA receptor in cultured hippocampal neurons [42].

In addition, previous results have shown that ISO acts as a positive allosteric modulator of GABA-A receptors [43]. These data suggest that the antinociceptive effect of ISO is, at least in part, due to the activation of GABA receptor subtypes, which has been demonstrated by the literature to have significant analgesic effects in neuropathic pain conditions [44]. Corroborating these data, studies have shown that atropine was able to reverse the anti-nociceptive action of ISO in the glutamate test [28]. These results lead us to the hypothesis that the antinociceptive effect of ISO may have a central origin, as the primary site of action for cholinomimetics in analgesia is the spinal cord. ISO likely activates muscarinic receptors in the spinal cord, resulting in increased release of inhibitory neurotransmitters (GABA) and decreased release of excitatory neurotransmitters (glutamate), and this activation may, in part, mediate its antinociceptive effect [28,45].

The pre-treatment of PTX-induced neuropathic pain animals with the glutamate NMDA receptor antagonist, MK-801, significantly enhanced the antinociceptive effect of ISO. The observed synergism between ISO and MK-801 suggests that the former substance may not be acting specifically on NMDA receptors and that its action could be indirect, potentially blocking steps in the signaling cascade of this neurotransmitter [46].

We assessed whether the release of [^3^H]GABA and [^14^C]Glutamate was affected by ISO directly on synaptosomes isolated from rat spinal cord in vitro. Firstly, to confirm the enrichment of synaptosomal preparations with nerve terminals and their greater capacity to release neurotransmitters such as GABA and Glutamate, we performed SDS-PAGE and Western blot analyses using total lysates (LTs) and synaptosomes (SNPs) of the rat spinal cord. The results show that SNP fractions were highly enriched in the synaptic vesicle protein, synaptophysin, vis a vis the content in the glial fibrillary acidic protein, GFAP (see Supplementary Materials), which was present in substantial amounts in spinal cord LTs.

After confirming the integrity of the cellular components present in SNP fractions, we proceeded to quantify the synchronous release of [^3^H]GABA and [^14^C]Glutamate upon synaptosomal depolarization by high KCl in the same samples. The results show that ISO favored the release of [^3^H]GABA from KCl-depolarized spinal cord synaptosomes. These data suggest that ISO, besides putatively activating muscarinic receptors in the spinal cord, may also act directly on nerve terminals to enhance GABA release. Braz et al. [47] further support this idea showing that the spinal transplantation of cortical precursors of GABAergic interneurons from the medial ganglionic eminence (MGE) could restore GABAergic signaling in the spinal cord to reverse allodynia and thermal hyperalgesia in neuropathic pain models induced by nerve injury or chemotherapy (PTX) in mice. The transplantation of cells with a deletion of the vesicular GABA transporter, which is necessary for storing synthesized GABA, had no effect on pain response, indicating that GABA released from these cells is crucial for regulating pain sensation.

Studies conducted in our laboratory have shown that ISO was able to reduce acute pain induced by formalin and capsaicin [28]. Substance P (SP), one of the neurotransmitters released in pain caused by capsaicin, induces glutamatergic synaptic transmission [48,49]. ISO, at doses of 3.12 and 6.25 mg/kg, significantly and dose-dependently inhibited nociception induced by glutamate in mice. These results suggest that ISO may act directly on glutamate receptors or prevent the release of various inflammatory mediators and neuropeptides involved in glutamate nociceptive transmission in the CNS and PNS. This indicates that ISO likely has both peripheral and central antinociceptive effects in this pain model [28,50,51].

In this context, we also evaluated the antinociceptive involvement of ISO in [^14^C]Glutamate release in vitro. Data confirmed the inhibitory action of ISO on depolarization-induced glutamate release from rat spinal cord synaptosomes. We also showed that that extracellular Ca^2+^ did not significantly affect this neurotransmitter release from depolarized synaptosomes of the spinal cord. Thus, the inhibitory action of this monoterpene may be related to a plasma membrane stabilization effect attenuating ion conductance and/or synaptic vesicles fusion to release glutamate, as predicted by the hypotonic hemolysis prevention in erythrocytes. Glutamate release inhibition may attenuate the post-synaptic activation of its receptors reducing pain transmission [50,51].

Given the inhibitory potential mediated by ISO, we analyzed whether it affected the concentration of the inflammatory pain mediating cytokine, TNF-α. Chemotherapy with PTX induces inflammatory responses through the release of brain-derived neurotrophic factors (BDNF) and inflammatory mediators. This leads to the chemotaxis and activation of macrophages in dorsal root ganglia (DRG), which combined with glial cells activation promote the release of inflammatory cytokines [52,53]. The most relevant cytokines observed in PTX-induced neuropathic pain are IL-1β, IL-8, and TNF-α [13], as well as IL-6, IL-4, and IL-10 [54]. One study showed an increase in the regulation of DRG and mRNA expression for pro-inflammatory cytokines (TNF-α and IL-1β) and neuropeptides, such as substance P and CGRP, in models of neuropathic pain [55].

Subacute treatment with ISO significantly reduced TNF-α levels in both sciatic nerves and sera of PTX-induced neuropathic animals. These results demonstrate that ISO may inhibit TNF-α production by cells or by acting at some point of its release pathway. These results agree with previous findings showing that anti-TNF-α agents or IL-1β receptor antagonists can attenuate PTX-induced neuropathic pain [55,56].

## 4. Material and Methods

### 4.1. Obtaining and Solubilizing ISO

Isopulegol (ISO) was purchased from Sigma-Aldrich. For in vivo experiments, ISO was solubilized using an aqueous vehicle containing 2% Tween 80 and 0.9% NaCl. For in vitro analysis, ISO was solubilized in the cell culture medium or in 5% dimethyl sulfoxide (DMSO).

### 4.2. Animals

Female Swiss mice (*Mus musculus*, 25 to 35 g) obtained from the Central Animal Facility of the Federal University of Piauí, Teresina, Piauí, were used and kept at a temperature of 24 ± 1 °C and a 12 h light/dark cycle with water and food ad libitum. All procedures related to the experimental protocols and euthanasia were submitted to and approved by the Animal Experimentation Ethics Committee (CEUA) of the Federal University of Piauí (740/2022; 13 October 2022) and ICBAS (Instituto de Ciências Biomédicas Abel Salazar), University of Porto (ORBEA/ICBAS-UP) (No. 224/2017). Experimental protocols were performed in accordance with the Brazilian law for the use of animals in research (Law number 11.794) and international guidelines on the care and use of experimental animals (Directive 2010/63/EU of the European Parliament and of the Council).

### 4.3. Evaluation of ISO Cytotoxicity

#### 4.3.1. Hypotonic Hemolysis with Rat Erythrocytes

To prepare the initial suspension of rat erythrocytes, 5 mL of blood was collected using a heparinized syringe (800 U/mL). An aliquot of 0.9 mL of blood was centrifuged for 15 min in a centrifuge tube at 1500 rpm. Subsequently, the plasma, as well as the leukocyte film on the surface of the red blood cell pellet, was extracted. An isotonic solution of 154 mM NaCl dissolved in 10 mM phosphate buffer (pH = 7) was added, for a total volume of 12.5 mL. The erythrocytes were resuspended by carefully inverting the tube several times. The hypotonic NaCl buffer that causes around 50% hemolysis was determined beforehand for the dilution of the drugs. ISO concentrations of 0.01, 0.03, 0.1, 0.3, 1, and 3 mM were used. The percentage of hemolysis obtained—relative hemolysis—was calculated from the total hemolysis value (100%) obtained from the absorbance value of the test tube containing only 10 mM phosphate buffer (pH = 7). Next, 4 mL of each solution was added to centrifuge tubes (in duplicate). An absolute hemolysis test tube, a relative hemolysis test tube, and a 0% hemolysis test tube (blank) were prepared by pipetting 10 mM phosphate buffer (pH = 7), hypotonic buffer selected from the previous test, and 154 mM NaCl buffer into 4 mL centrifuge tubes.

Subsequently, 0.2 mL of the initial erythrocyte suspension was added to all the tubes with the respective solutions. Each tube was shaken gently in a vortex and left to stand for 10 min (hemolysis time). At the end of the hemolysis time, the tubes were centrifuged at 3000 rpm for 15 min. The supernatants were separated into Spectronic tubes, and the absorbance was read at a wavelength of 540 nm on a Spectronic 6305 spectrophotometer (Jenway, model 6305, Tokyo, Japan). Calculations were performed as follows: % absolute hemolysis = absorbance of sample × 100/absorbance of phosphate buffer; relative hemolysis = % absolute hemolysis of the sample/% absolute hemolysis of the hypotonic NaCl buffer.

#### 4.3.2. Evaluation of the Cytotoxic Activity of ISO

Human glioblastoma tumor cell lines (U87), sourced from the American Type Culture Collection (ATCC), were provided by Prof. Dr. Miriam Teresa Paz Lopes (Department of Pharmacology, Federal University of Minas Gerais, Brazil). The cells were grown in DMEM medium (GIBCO, Baltimore, MD, USA) and supplemented with 10% fetal bovine serum (Cultilab, Brazil) and an antibiotic solution containing penicillin and streptomycin (10,000 U/mL—GIBCO, Baltimore, MD, USA). The cells were kept in a culture oven (Shel Lab^®^, Cornelius, OR, USA) at 5% CO_2_, 95% humidity, and 37 °C. When cell confluence reached approximately 90%, the cells were enzymatically dissociated with 0.5% (*w*/*v*) trypsin diluted in RPMI 1640, and subcultures were carried out for subsequent tests.

The cytotoxicity of paclitaxel was assessed using the MTT assay (3-[4,5-dimethylthiazol-2yl]-2,5-diphenyl-2H bromine tetrazolate). The cell lines (MCF-7, MDA-MB-231, and MCF10) were seeded in 96-cavity plates (5 × 10^3^ cells/cavity) and kept in an oven for 24 h for adhesion. The cytotoxic effect of ISO was assessed using the resazurin method [57].

Thus, U87 cells seeded at a density of 4 × 10^3^ cells/cavity were exposed to ISO at concentrations of 0.9756625 to 500 μg/mL for 72 h. Then, 20 μL of resazurin at 60 μg/mL was added and, after 4 h, fluorescence was determined using a microplate reader (GloMax Microplate Reader, Promega, Madison, WI, USA), using the wavelength of 520 nm for excitation and 580 nm for emission.

The results of these tests were expressed as % cell viability, considering the untreated group as 100%. The mean inhibitory concentration (IC50) values were determined from the non-linear regression of the cell viability curve using GraphPad Prisma 8 software (San Diego, CA, USA). Each experiment was performed in triplicate and repeated three times to ensure consistent results.

#### 4.3.3. Evaluation of the Effect of ISO Alone or in Combination with Paclitaxel on the Viability of Human Breast Carcinoma Cells

The interaction between PTX and ISO was evaluated in order to ensure that the antiproliferative effect of ISO overrides the cytotoxic action of PTX. For this purpose, it was decided to use MDA-MB-231 human breast carcinoma cells (negative for the expression of estrogen, progesterone, and epidermal growth factor receptors), because they are resistant to PTX. The MDA-MB-231 cells (3 × 10^3^/cavity) were seeded in 96-well plates and incubated for 72 h at 37 °C in a 5% CO_2_ oven. After this time, the cells were treated with PTX, alone or in combination with ISO, at a fixed ratio, equivalent to the respective IC50 values (which include CI50 × 0.25, ×0.5, ×1, ×2, and ×4), for 48 h. The combination concentrations of PTX and ISO, free or associated, were chosen based on the method proposed by Bijnsdorp, Giovannetti, and Peters [58], which establishes that two equidistant concentrations should be tested, above and below the IC50, in a 1:10 ratio of PTX to ISO.

The classification of the pharmacological interaction between paclitaxel and ISO was based on the method of [59], where the combination index (CI) and the fraction of affected cells (Fa) for viability were estimated using a specific statistical program for analyzing combinations of compounds, CalcuSyn v. 2.0 (Biosoft). The ratio between CI and Fa provides a quantitative definition for additive effect (CI between 0.8 and 1.2), synergism (CI < 0.8), and antagonism (CI > 1.2) in drug combinations [58].

### 4.4. Determining Nociception by Mechanical Stimulation and Thermal Sensitivity to Cold

The animals were submitted to mechanical nociceptive threshold assessment using von Frey filaments. The animals were acclimatized for at least 30 min before the behavioral tests. The mechanical nociceptive threshold was measured on a transparent acrylic platform (9 × 7 × 11 cm) to allow access to the ventral surface of the hind paws. The frequency of paw withdrawals was obtained through three applications of von Frey filaments (a stimulus of 1 s each) with growing intensity of weight (0.04–15 g). All groups of animals were previously evaluated (baseline) at different times after the induction of neuropathy [60].

The acetone test was used to assess thermal sensitivity to cold (10 °C). An aliquot of 0.05 mL of acetone was dropped onto the ventral surface of the animal’s paw through the fenestrated floor. After correctly wetting an animal’s paw with acetone, animals were observed during a period of 60 s for the following responses: a rapid paw withdrawal, paw flicking and paw licking or jumping, grooming, and abnormal agitation. The durations of these responses were recorded for each group [61]. This test was performed to assess both acute and subacute thermal sensitivity induced by paclitaxel.

#### 4.4.1. Paclitaxel-Induced Neuropathy

**Figure 8:**
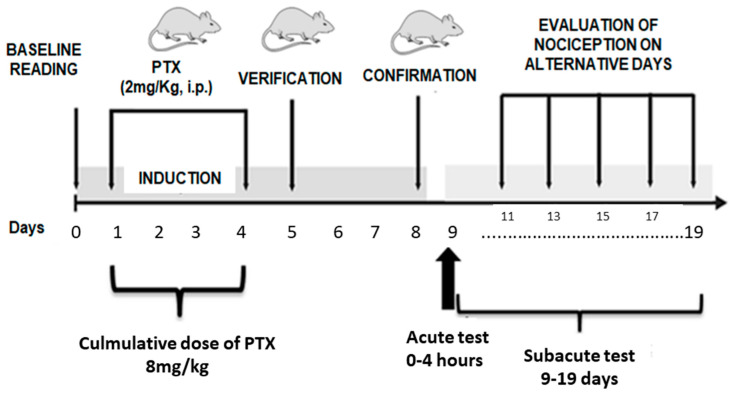
Diagram of the induction of neuropathy with the specific days of the behavioral evaluations.

Animals received 2 mg/kg of paclitaxel (PTX) intraperitoneally diluted in saline solution for four consecutive days, resulting in a cumulative dose of 8 mg/kg, as shown in Figure 8. The animals in the sham group were not treated with paclitaxel or any other treatment (adapted from [62]).

#### 4.4.2. Acute Effect of ISO in Paclitaxel-Induced Neuropathy

On the ninth day (five days after the end of paclitaxel administration), the animals underwent mechanical (von Frey) and thermal (acetone test) evaluation at time intervals of 0, 1, 2, 3, and 4 h after administration of ISO. Significant differences between the means were assessed in tests before and after induction with PTX. The animals were divided into groups (n = 6) and treated with ISO (at doses of 6.25, 12.5, 25, and 50 mg/kg, v.o.), vehicle (2% Tween 80 and 0.9% NaCl, v.o.), and morphine (10 mg/kg, s.c.), along with a sham group (non-neuropathic untreated animals).

#### 4.4.3. Subacute Effect of ISO in Paclitaxel-Induced Neuropathy

On the day of treatment after assessment of the acute effect of ISO (previous session), animals were treated daily with ISO (at doses of 6.25, 12.5, 25, and 50 mg/kg, v.o.), vehicle (2% Tween 80 and 0.9% NaCl, v.o.), and duloxetine (30 mg/kg, s.c.). Mechanical sensitivity (von Frey) and cold sensitivity (acetone test) assessment were performed on days 8, 9, 12, 15, 18, and 19 of the experimental protocol.

#### 4.4.4. Evaluation of the Participation of the GABAergic System in the Antinociceptive Effect of ISO

On day 9 of the experimental protocol, neuropathic animals were pre-treated with flumazenil (5 mg/kg, i.p.), a competitive inhibitor at the benzodiazepine recognition site of the GABA-A receptor. After 30 min, the animals received ISO (25 mg/kg, v.o.). Neuropathic animals treated with vehicle solution (0.9% NaCl + 2% Tween 80, v.o.) or ISO alone were used as controls. After 1 h, the animals were assessed for mechanical sensitivity (von Frey filaments).

#### 4.4.5. Evaluation of Participation of NMDA Receptors in the Antinociceptive Effect of ISO

On day 9 of the experimental protocol, neuropathic animals were pre-treated with MK-801 (0.03 mg/kg, i.p.), a specific, selective, and non-competitive NMDA receptor antagonist. After 30 min, the animals were given ISO (25 mg/kg, v.o.). Neuropathic animals treated with vehicle solution (0.9% NaCl + 2% Tween 80, v.o.) or treated with ISO alone were used as controls. After 1 h, the animals were assessed for nociception induced by mechanical stimulation (von Frey).

#### 4.4.6. Determination of TNF-α Levels by ELISA Assay

Assessments of tissue levels of interleukins were carried out after the end of the subacute treatment, wherein the animals were euthanized, and blood samples as well as sciatic nerves of animals were collected and processed for analysis. The enzyme-linked immunosorbent assay was performed with the serum and sciatic nerve of neuropathic animals treated with ISO. The test was conducted using 100 μL of each component of the reaction. TNF-α was assessed using a standard ELISA kit (R&D Systems, Inc., Minneapolis, MN, USA) according to the manufacturer’s instructions. The results on TNF-α were expressed as ug/mL of blood samples or sciatic nerve tissue.

### 4.5. Evaluation of GABA and Glutamate Release from Spinal Cords in Vitro

#### 4.5.1. Isolation of the Spinal Cord

Spinal cords were taken from healthy female rats (n = 4 animals), dissected, and gently homogenized in cold oxygenated Krebs solution (95% O_2_ and 5% CO_2_) (in mM: glucose 5.5, NaCl 136, KCl 3, MgCl_2_ 1.2, Na_2_ HPO_4_ 1.2, NaHCO_3_ 16.2, CaCl_2_ 0.5; pH 7.40). The homogenates were filtered with a nylon filter (mesh size 100 μm). The filtrate was left to stand for 30–45 min until a pellet was formed, which was resuspended in Krebs solution and left at room temperature. The protein concentration determined by the bicinchoninic acid method (Pierce bicinchoninic acid protein assay^TM^, Thermo Scientific, Rockford, IL, USA) was adjusted to 6.25 mg protein mL^−1^. Synaptosomes obtained from the spinal cords of female rats were used to quantify the release of [^3^H]GABA and [^14^C]glutamate.

SDS-PAGE and Western blotting analyses were carried out on total lysate (LT) and synaptosomes (SNPs) from the spinal cords of different animals to verify the enrichment in nerve terminals of synaptosome preparations isolated from spinal cords and their ability to release the neurotransmitters GABA and glutamate. Total spinal cord membrane lysates (LT) and synaptosomes (SNPs) (n = 4) from healthy rats were homogenized in radio-immunoprecipitation assay (RIPA) buffer containing 25 mM Tris-HCl (pH 7.6), 150 mM NaCl, 1% sodium deoxycholate, 1% triton-X-100, 0.1% SDS, 5 mM EDTA, and a protease inhibitor cocktail (Sigma-Aldrich, St. Louis, MO, United States). The protein content of the samples (30 mg) was assessed using the BCA method. The samples were solubilized at 70 °C in SDS reduction buffer (125 mM Tris-HCl (pH 6.8), 4% SDS, 0.005% bromophenol blue, 20% glycerol, and 5% 2-mercaptoethanol) for 10 min, electrophoresed in 12.5% SDS-polyacrylamide gels, and electrotransferred to PVDF membranes (Merck MilliPore, Temecula, CA, USA). The membranes were blocked for 1 h in Tris-buffered saline (TBS; in mM: Tris—HCl 10 (pH 7.6), NaCl 150) containing 0.05% Tween 20 and 5% BSA and subsequently incubated overnight at 4 °C with primary antibodies: mouse anti-synaptophysin (1:1000, Chemicon, Temecula, CA, USA) and mouse anti-GFAP (1:500, Chemicon, Temecula, CA, USA). The membranes were washed three times for 10 min in 0.05% Tween 20 in TBS and then incubated with peroxidase-conjugated anti-rabbit or anti-mouse secondary antibodies for 120 min at room temperature. The antigen–antibody complexes were visualized by chemiluminescence with the Immun-Star WesternC kit (Bio-Rad Laboratories, Hercules, CA, USA) using the ChemiDoc MP imaging system (Bio-Rad Laboratories, Hercules, CA, USA). Gel band image densities were quantified using ImageJ software® (version 1.8.0, US National Institutes of Health, MD, USA).

#### 4.5.2. Quantification of [^3^H]GABA and [^14^C]Glutamate Release from KCl-Depolarized Synaptosomes of the Rat Spinal Cord

Spinal cord synaptosomes were incubated with [^3^H]GABA (0.25 μCi mL^−1^; 70 Ci mmol^−1^; 0.5 μM) and [^14^C]Glutamate (0.25 μCi mL^−1^; 0.270 Ci mmol^−1^; 10 μM) for 10 min at 37 °C, and their release was measured simultaneously. Aliquots of synaptosome suspension from the spinal cords were placed on glass fiber filters (Merck Millipore, Cork, Ireland), which were mounted in 365 μL chambers of a semi-automatic 12-sample superfusion system (SF-12 Superfusion 1000, Brandel, Gaithersburg, MD, USA). The filters containing the samples were superfused at a rate of 0.5 mL min^−1^, at 37 °C, with a physiological solution containing (in mM): 128 NaCl, 1.2 MgCl_2_, 3 KCl, 10 glucose, 10 HEPES–Na (pH = 7.4), 2.2 CaCl_2_, and 0.1 aminooxyacetic acid, an inhibitor of GABA degradation in the tissue by 4-aminobutyrate aminotransferase (GABA-T). After an equilibration period of 26 min, fractions of 2 min were automatically collected using the SF-12 superfusion system. Eight (S1) and twenty-six (S2) min after the start of sampling, the spinal cords were depolarized with a solution containing high KCl (15 mM) for 2 min; this was done by changing the inlet tube from one vial to another containing the depolarizing agent. High KCl is the most common strategies for depolarizing the spinal cord plasma membrane, as it allows differentiation between Na^+^-channel-mediated presynaptic responses.

Isopulegol was added 15 min before S2. The radioactive content of the collected fractions and the remainder on the filters at the end of the protocol were measured by liquid scintillation spectrometry (TriCarb2900TR, Perkin Elmer, Boston, MA, USA).

### 4.6. Statistical Analysis

The results obtained from the behavioral tests were expressed as the mean ± standard error of the mean SEM. A two-way analysis of variance (ANOVA) was performed for the acute and subacute tests and a one-way analysis of variance for the analysis of mechanism of action, followed by a Tukey or Bonferroni post hoc test for two-way analyses and Tukey post hoc test for one-way analyses. Results were considered statistically significant when * *p* < 0.05. All analyses were conducted using GraphPad Prism software (Graphstats Technologies) version 8.0.

## 5. Conclusions

These findings suggest that ISO has acute and subacute antinociceptive activity in response to different stimuli (mechanical and thermal/cold) in an animal model of neuropathic pain induced by PTX. Part of this effect may be due to activation of inhibitory GABAergic pathways (including promotion of GABA release and, subsequent, GABA receptors stimulation), prevention of glutamatergic pathways (either involving inhibition of glutamate release and/or blockage of NMDA receptor activation), and stabilization of neuronal plasma membranes. All these features may foster ISO to re-equilibrate unbalanced neuropathic neurotransmission caused by PTX by favoring GABA-mediated inhibition over glutamatergic excitation. Additionally, ISO on its own exhibited anti-inflammatory effects by reducing the concentration of TNF-α both in the sciatic nerve and in the serum. ISO proved to be a low toxicity compound, both for U87 neuronal cells and MDA-MB-231 Human breast carcinoma cells, at the studied concentrations. ISO association with PTX did not preclude the cytotoxic effect of PTX against human breast cancer cells and even produced a synergic anti-neoplastic effect. Therefore, the pharmacological potential of ISO demonstrated in this study provides the basis for its use as a prototype in the development of drugs aimed at treating neuropathic pain, while promoting the anti-proliferative effect of chemotherapies.

## Data Availability

All of the data generated or analyzed during this study are included within this article and Supplementary Materials file; further inquiries can be directed to the corresponding author.

## Supplementary Materials

The following supporting information can be downloaded at https://www.mdpi.com/article/10.3390/ph18020256/s1: The results of SNP fractions.

## References

[1] Raja S.N., Carr D.B., Cohen M., Finnerup N.B., Flor H., Gibson S., Keefe F.J., Mogil J.S., Ringkamp M., Sluka K.A. (2020). The revised International Association for the Study of Pain definition of pain: Concepts, challenges, and compromises. Pain.

[2] Alles S.R.A., Smith P.A. (2018). Etiology and Pharmacology of Neuropathic Pain. Pharmacol. Rev..

[3] Gao T.C., Wang C.H., Wang Y.Q., Mi W.L. (2023). IL-33/ST2 Signaling in the Pathogenesis of Chronic Pain and Itch. Neuroscience.

[4] Rangel O., Telles C. (2012). Treatment of cancer pain in palliative care. Rev. Hosp. Univ. Pedro Ernesto.

[5] Lakkad M., Martin B., Li C., Harrington S., Dayer L., Painter J.T. (2023). Factors Associated with Guideline-Concordant Pharmacological Treatment for Neuropathic Pain Among Breast Cancer Survivors. Clin. Breast Cancer.

[6] Jheng Y.-W., Chan Y.-N., Wu C.-J., Lin M.-W., Tseng L.-M., Wang Y.-J. (2024). Neuropathic Pain Affects Quality of Life in Breast Cancer Survivors with Chemotherapy-Induced Peripheral Neuropathy. Pain Manag. Nurs..

[7] Zukas A.M., Schiff D. (2018). Neurological complications of new chemotherapeutic agents. Neuro Oncol..

[8] Borkowska H.A., Bagues A., Tu L., Liu J.Y.H., Lu Z., Rudd J.A., Nurgali K., Abalo R. (2022). Mechanisms of chemotherapy-induced neurotoxicity. Front. Pharmacol..

[9] Lustberg M.B., Kuderer N.M., Desai A., Bergerot C., Lyman G.H. (2023). Mitigating Late and Long-Term Adverse Events Associated with Cancer Treatment: Implications for Survivorship. Nat. Rev. Clin. Oncol..

[10] Xiaoman M., Wu S., Huang D., Li C. (2024). Complications and Comorbidities Associated with Antineoplastic Chemotherapy: Rethinking Drug Design and Delivery for Anticancer Therapy. Acta Pharm. Sin. B.

[11] Rivera E., Cianfrocca M. (2015). Overview of Neuropathy Associated with Taxanes for the Treatment of Metastatic Breast Cancer. Cancer.

[12] Timmins H.C., Li T., Trinh T., Kiernan M.C., Harrison M., Boyle F., Friedlander M., Goldstein D., Park S.B. (2021). Weekly Paclitaxel-Induced Neurotoxicity in Breast Cancer: Outcomes and Dose Response. Oncologist.

[13] Klein I., Lehmann H.C. (2021). Pathomechanisms of Paclitaxel-Induced Peripheral Neuropathy. Toxics.

[14] Staff N.P., Grisold W., Grisold A., Kamerman D., Ahlawat S., Gibbons C.H., Lomen-Hoerth C., Reichard R., Thomas R., Taylor J.A. (2020). Pathogenesis of Paclitaxel-Induced Peripheral Neuropathy: A Current Review of In Vitro and In Vivo Findings Using Rodent and Human Model Systems. Exp. Neurol..

[15] Thouaye M., Yalcin I. (2023). Neuropathic Pain: From Actual Pharmacological Treatments to New Therapeutic Horizons. Pharmacol. Ther..

[16] Pina L.T.S., Guimarães A.G., Santos W.B.D.R., Oliveira M.A., Rabelo T.K., Serafini M.R. (2021). Monoterpenes as a Perspective for the Treatment of Seizures: A Systematic Review. Phytomedicine.

[17] Prerna P., Chadha J., Khullar L., Mudgil U., Harjai K. (2024). A Comprehensive Review on the Pharmacological Prospects of Terpinen-4-ol: From Nature to Medicine and Beyond. Fitoterapia.

[18] Mansi V.G., Sahu B.D. (2024). Carvacrol and Its Effect on Cardiovascular Diseases: From Molecular Mechanism to Pharmacological Modulation. Food Biosci..

[19] Vernin G.A., Parkanyi C., Cozzolino F., Fellous R. (2004). GC/MS Analysis of Volatile Constituents of *Corymbia citriodora* Hook on Réunion. J. Essent. Oil Res..

[20] Paik S.Y., Kok K.H., Bico S.M. (2005). Essential Oils of Zanthoxylum schinifolium Pericarp Induce Apoptosis in Human Hepatoma HepG2 Cells via Increased Reactive Oxygen Species Production. Biol. Pharm. Bull..

[21] Vane J.R., Botting R.M. (1995). A Better Understanding of Anti-Inflammatory Drugs Based on Isoforms of Cyclooxygenase (COX-1 and COX-2). Adv. Prostaglandin Thromboxane Leukot. Res..

[22] Sreelekshmi R., Latha P.G., Arafat M.M., Shyamal S., Shine V.J., Anuja G.I., Suja S.R., Rajasekharan S. (2007). Anti-Inflammatory, Analgesic and Anti-Lipid Peroxidation Studies on Stem Bark of *Ficus religiosa* Linn. Nat. Prod. Radiance.

[23] Blumenthal M., Goldberg A., Brinckmann J. (2000). Herbal Medicine—Expanded Commission E Monographs.

[24] Silva M.I.G., de Aquino Neto M.R., Neto P.F.T., Moura B.A., Amaral J.F.D., de Sousa D.P., Vasconcelos S.M.M., de Sousa F.C.F. (2007). Central Nervous System Activity of Acute Isopulegol Administration in Mice. Pharmacol. Biochem. Behav..

[25] Silva M.I.G., Silva M.A.G., Aquino-Neto M.R.D., Moura B.A., Sousa H.L.D., Lavor E.P.H.D., Vasconcelos P.F.D., Macêdo D.S., Sousa D.P.D., Vasconcelos M.S.M. (2007). Effects of Isopulegol on Seizures Induced by Pentylenetetrazol in Rats: Possible Involvement of the GABAergic System and Antioxidant Activity. Phytotherapy.

[26] Silva M.I.G., Moura B.A., Aquino-Neto M.R., Tomé A.R., Rocha N.F.M., Carvalho A.M.R., Macêdo D.S., Vasconcelos S.M.M., Sousa D.P., Viana G.S.D.B. (2009). Gastroprotective Activity of Isopulegol on Experimentally Induced Gastric Lesions in Mice: Investigation of Possible Mechanisms of Action. Naunyn-Schmiedeberg’s Arch. Pharmacol..

[27] Ramos A.G.B., de Menezes I.R.A., da Silva M.S.A., Torres Pessoa R., de Lacerda Neto L.J., Rocha Santos Passos F., Melo Coutinho H.D., Iriti M., Quintans-Júnior L.J. (2020). Antiedematogenic and Anti-Inflammatory Activity of the Monoterpene Isopulegol and Its β-Cyclodextrin (β-CD) Inclusion Complex in Animal Inflammation Models. Foods.

[28] Próspero D.F.A., Filho A.C.R., Piauilino C.A., Lopes E.M., de Sousa D.P., de Castro Almeida F.R. (2018). Effects of Isopulegol in Acute Nociception in Mice: Possible Involvement of Muscarinic Receptors, Opioid System and L-Arginine/NO/cGMP Pathway. Chem. Biol. Interact..

[29] Guimarães A.G., Quintans J.S.S., Quintans-Júnior L.J. (2014). Monoterpenes with Analgesic Activity—A Systematic Review. Phytother. Res..

[30] Gouveia D.N., Guimarães A.G., Oliveira M.A., Rabelo T.K., Pina L.T.S., Santos W.B.R., Almeida I.K.S., Andrade T.A., Serafini M.R., Lima B.S. (2023). Nanoencapsulated α-Terpineol Attenuates Neuropathic Pain Induced by Chemotherapy Through Calcium Channel Modulation. Polymer Bull..

[31] Pinheiro-Neto F.R., Lopes E.M., Acha B.T., Gomes L.D.S., Dias W.A., Reis Filho A.C.D., Leal B.S., Rodrigues D.C.D.N., Silva J.D.N., Dittz D. (2021). α-Phellandrene Exhibits Antinociceptive and Tumor-Reducing Effects in a Mouse Model of Cancer Pain. Toxicol. Appl. Pharmacol..

[32] Alqahtani A., Abdelhameed M.F., Abdou R., Ibrahim A.M., Dawoud M., Alasmari S.M., El Raey M.A., Attia H.G. (2023). Mechanistic Action of Linalyl Acetate: Acyclic Monoterpene Isolated from Bitter Orange Leaf as Anti-Inflammatory, Analgesic, Antipyretic Agent: Role of TNF-α, IL-1β, PGE2, and COX-2. Ind. Crops Prod..

[33] Bhatia S.P., McGinty D., Letizia C.S., Api A.M. (2008). Fragrance Material Review on isopulegol. Food Chem. Toxicol..

[34] Yang H., Bai J., Ma C., Wang L., Li X., Zhang Y., Xu Y., Yang Y. (2020). Degradation models, structure, rheological properties and protective effects on erythrocyte hemolysis of the polysaccharides from *Ribes nigrum* L.. Int. J. Biol. Macromol..

[35] Bráz J.M., Sharif-Naeini R., Vogt D., Kriegstein A., Alvarez-Buylla A., Rubenstein J.L., Basbaum A.I. (2012). Precursors of GABAergic neurons from the forebrain integrate into the adult spinal cord and reduce injury-induced neuropathic pain. Neuron.

[36] Ramos J.O.C.A., Santos D.G., Santana D.A.S., Alves S.M., Thomazzi S. (2013). Chemical constituents and potential anti-inflammatory activity of the essential oil of *Croton argyrophyllus* leaves. Rev. Bras. Farmacogn..

[37] Serra S., Brenna E., Fuganti C., Maggioni F. (2003). Lipase-catalyzed resolution of p-menthan-3-ols monoterpenes: Preparation of the enantiomer-enriched forms of mentol, isopulegol, trans- and cis-piperitol, and cis-isopiperitol. Tetrahedron Asymmetry.

[38] Brid T., Miranda G., Lozano B., Baamonde A. (2015). Topical L-menthol for Postradiotherapy Neuropathic Pain: A Case Report. J. Pain Symptom Manag..

[39] Li C., Lei Y., Tian Y., Xu S., Shen X., Wu H., Bao S., Wang F. (2019). The etiological contribution of GABAergic plasticity to the pathogenesis of neuropathic pain. Mol. Pain.

[40] Malan T.P., Mata H.P., Porreca F. (2002). Spinal GABA(A) and GABA(B) receptor pharmacology in a rat model of neuropathic pain. Anesthesiology.

[41] Pigott T., McPeak A., de Chastelain A., DeMayo M.M., Rasic N., Rayner L., Noel M., Miller J.V., Harris A.D. (2023). Changes in Brain GABA and Glutamate and Improvements in Physical Functioning Following Intensive Pain Rehabilitation in Youth with Chronic Pain. J. Pain.

[42] Zhang X.B., Jiang P., Gong N., Hu X.L., Fei D., Xiong Z.Q., Xu L., Xu T.L. (2008). GABA type A receptor as a central target of the TRPM8 agonist menthol. PLoS ONE.

[43] Watt E.E., Betts B.A., Kotey F.O., Humbert D.J., Griffith T.N., Kelly E.W., Veneskey K.C., Gill N., Rowan K.C., Jenkins A. (2008). Menthol shares general anesthetic activity and sites of action on the GABA(A) receptor with the intravenous agent, propofol. Eur. J. Pharmacol..

[44] Juarez-Salinas D.L., Ribeiro A., Etlin S., Pô V., Sohal I.A., Basbaum A.I. (2019). GABAergic cell transplants into the anterior cingulate cortex reduce neuropathic pain aversion. Brain.

[45] Qian X., Zhao X., Yu L., Yin Y., Zhang X.-D., Wang L., Li J.-X., Zhu Q., Luo J.-L. (2023). Current status of GABA receptor subtypes in analgesia. Biomed. Pharmacother..

[46] Song X., Jensen M.Ø., Jogini V., Stein R.A., Lee C.H., Mchaourab H.S., Shaw D.E., Gouaux E. (2018). Mechanism of NMDA receptor channel block by MK-801 and memantine. Nature.

[47] Braz J.M., Oliveira Z., Oliveira J.L., Rubenstein J.L., Basbaum A.I. (2015). Transplant-mediated increase of GABAergic spinal cord inhibition reverses paclitaxel-induced mechanical and thermal hypersensitivity. Pain.

[48] Wallengren J., Hakanson R. (1987). Effects of substance P, neurokinin A and calcitonin gene-related peptide in human skin and their involvement in sensory-mediated responses. Eur. J. Pharmacol..

[49] Sakurada T., Katsumata K., Tan-No S., Sakurada S., Kisara K. (1992). The capsaicin test in mice for evaluating tachykinin antagonists in the spinal cord. Neuropharmacology.

[50] Millan M.J. (1999). The induction of pain: An integrative review. Prog. Neurobiol..

[51] Fundytus M.E. (2001). Glutamate receptors and nociception: Implications for the drug treatment of pain. CNS Drugs.

[52] Sisignano M., Baron R., Scholich K., Geisslinger G. (2014). Mechanism-based treatment for chemotherapy-induced peripheral neuropathic pain. Nat. Rev. Neurol..

[53] Carozzi V.A., Canta A., Chiorazzi A. (2015). Chemotherapy-induced peripheral neuropathy: What do we know about mechanisms?. Neurosci. Lett..

[54] Ha J.W., You M.J., Park H.S., Kim J.W., Kwon M.S. (2019). Differential effect of LPS and paclitaxel on microglial functional phenotypes and circulating cytokines: The possible role of CX3CR1 and IL-4/10 in blocking persistent inflammation. Arch. Pharm. Res..

[55] Balkrishna A., Karumuri S., Sakat S.S., Haldar S., Varshney A. (2022). Anti-oxidant profile of Divya-Peedantak-Vati abates paclitaxel-induced hyperalgesia and allodynia in CD-1 mice model of neuropathic pain. Phytomedicine Plus.

[56] Clark A.K., Old E.A., Malcangio M. (2013). Neuropathic pain and cytokines: Current perspectives. J. Pain Res..

[57] Czekanska E.M. (2011). Evaluation of Cell Proliferation Using Resazurin-Based Fluorescent Dye. Methods Mol. Biol..

[58] Bijnsdorp I.V., Giovannetti E., Peters G.J. (2011). Analysis of Drug Interactions. Cancer Cell Cult. Methods Protoc..

[59] Chou T.C. (2010). Drug Combination Studies and Their Synergy Quantification Using the Chou-Talalay Method. Cancer Res..

[60] Chaplan S.R., Bach F.W., Pogrel J., Robbins E., Weber M., Schupp C., Gopalakrishnan M. (1994). Quantitative Assessment of Tactile Allodynia in the Rat Paw. J. Neurosci. Methods.

[61] Flatters S.J.L., Bennett G.J. (2004). Ethosuximide Reverses Paclitaxel- and Vincristine-Induced Painful Peripheral Neuropathy. Pain.

[62] Qabazard B., Masocha W., Khajah M., Philips O.A. (2020). H_2_S donor GYY4137 ameliorates paclitaxel-induced neuropathic pain in mice. Biomed Pharmacother..

