# Supplementary material for: Anti-Hyperalgesic Effect of Isopulegol Involves GABA and NMDA Receptors in a Paclitaxel-Induced Neuropathic Pain Model
Source: Pharmaceuticals (Basel). 2025 Feb 14;18(2):256. doi: 10.3390/ph18020256 (PMC11860001; doi:10.3390/ph18020256)
Supplement: Supplementary file 1 [file pharmaceuticals-18-00256-s001.zip › pharmaceuticals-3405861-supplementary.pdf]

SUPPLEMENTARY MATERIALS

1. MATERIAL AND METHODS

1.1 SDS - PAGE and Western Blot analysis

Rat spinal cord total membrane lysates were homogenized in Immunoprecipitation Assay (RIPA) buffer containing: 25 mM Tris-HCl (pH 7.6), 150 mM NaCl, 1% sodium deoxycholate, 1% triton-X-100, 0.1% SDS, 5 mM EDTA and a protease inhibitor cocktail (Sigma-Aldrich, St. Louis, MO, United States). The protein content of the samples was assessed using the BCA method. The samples were solubilized at 70°C in SDS reduction buffer [125 mM Tris-HCl (pH 6.8), 4% SDS, 0.005% bromophenol blue, 20% glycerol and 5% 2-mercaptoethanol] for 10 min, electrophoresed on 12.5% SDS-polyacrylamide gels and electrotransferred to PVDF membranes (Merck MilliPore, Temecula, CA, United States). The membranes were blocked for 1 h in Tris-buffered saline [TBS; in mM: Tris - HCl 10 (pH 7.6), NaCl 150] containing Tween 20 0. 05% and BSA 5% and subsequently incubated overnight at 4°C with primary antibodies: mouse anti-synaptophysin (1:1000, Chemicon, Temecula, CA, United States) and mouse anti-GFAP (1:500, Chemicon, Temecula, CA, United States). The membranes were washed three times for 10 min in 0.05% Tween 20 in TBS and then incubated with peroxidase-conjugated anti-rabbit or anti-mouse secondary antibodies for 120 min at room temperature. The antigen-antibody complexes were visualized by chemiluminescence with the Immun-Star WesternC kit (Bio-Rad Laboratories, Hercules, CA, United States) using the ChemiDoc MP imaging system (Bio-Rad Laboratories, Hercules, CA, United States). Gel band image densities were quantified with Image J (National Institute of Health, United States).

2. RESULTS

2.1 SDS - PAGE and Western Blotting analysis

SDS-PAGE and Western Blotting analyses were carried out on the total-LT lysate and the synaptosome-SNPs from the spinal cords to verify the enrichment in nerve terminals of the synaptosome preparations isolated from the spinal cords and their greater capacity to release neurotransmitters such as GABA and Glutamate. The results suggest the presence of glial fibrillary acidic protein (GFAP) and synaptophysins (SYN) in both LT (86.7% and 13.3%, respectively) and SNPs (GFAP-42.6% and SYN- 57.3%), confirming that the handling of synaptosomes preserved the presence of synaptic cells and vesicles, where neurotransmitters are stored, as shown below.

86,7%

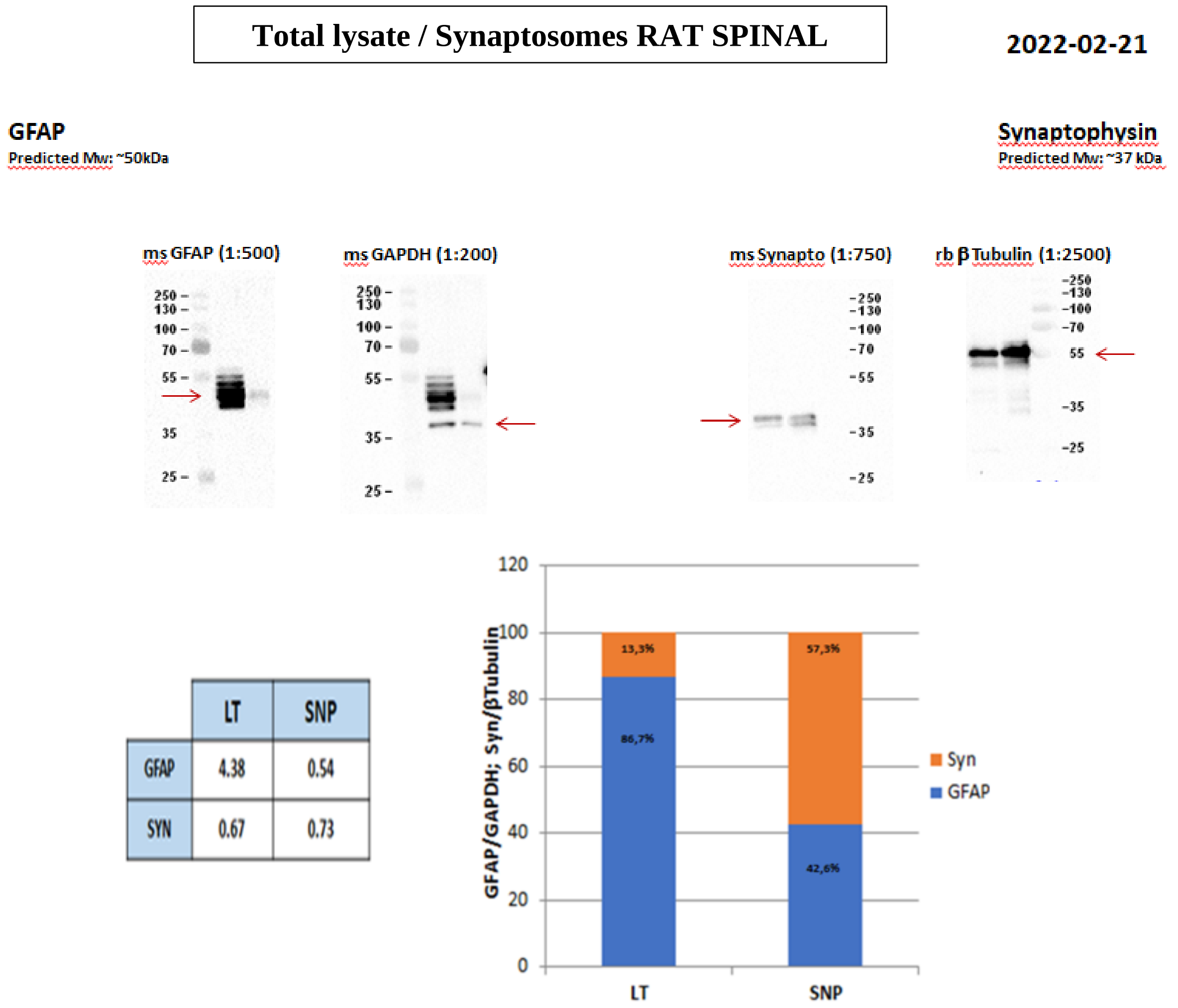

	LT	SNP
GFAP	4.38	0.54
SYN	0.67	0.73

Figure S1: PAGE and Western Blotting analysis of total lysate (TL) and synaptosomes (SNP) from rat spinal cord. *GFAP: Glial Fibrillary Acid Protein, *SYN: Synaptophysin.

3. DISCUSSION

In order to prove the enrichment in nerve terminals of synaptosome preparations isolated from rat spinal cords, and their greater capacity to release neurotransmitters such as GABA and Glu, SDS-PAGE and Western Blotting analyses were carried out using total lysates - LTs and synaptosomes - SNPs. The results show that the SNPs had a higher relative density of the synaptophysin protein present in the synaptic vesicles when compared to the glial protein, GFAP, contrary to what was found in the LTs where contamination with glial cells is higher.

GFAP is a protein of the intermediate filamentary cytoskeleton expressed in astrocytes; increased blood and cerebrospinal fluid (CSF) concentrations of GFAP have also been reported in neuronal diseases such as multiple sclerosis, and may be associated with a progressive course of the disease (AMMITZBOLL et al., 2023). Synaptophysin is the most abundant synaptic vesicle protein by mass and regulates the endocytosis of synaptic vesicles. Synaptophysin is a commonly used

immunohistochemical marker with high sensitivity and limited specificity, especially in high-grade CNS tumors (RIA UHLIG et al. 2022).

After confirming the integrity of the cellular components present in the synaptosomes, we moved on to quantifying the release of the neurotransmitter [3H] GABA....

MINISTÉRIO DA EDUCAÇÃO
UNIVERSIDADE FEDERAL DO PIAUÍ
PRÓ-REITORIA DE PESQUISA E INOVAÇÃO
COMISSÃO DE ÉTICA NO USO DE ANIMAIS

CERTIFICADO

Certificamos que a proposta intitulada "***Investigação da atividade antinociceptiva do isopulegol, terpinoleno e óxido de rosa em modelos animais de dor neuropática***", registrada sob o nº **740/2022**, sob a responsabilidade da **Profa. Dra. FERNANDA REGINA DE CASTRO ALMEIDA do Departamento de Bioquímica e Farmacologia/CCS/UFPI** que envolve a produção, manutenção ou utilização de animais pertencentes ao filo Chordata, subfilo Vertebrata (exceto humanos), para fins de Pesquisa Científica, encontra-se de acordo com os preceitos da Lei nº 11.794, de 8 de outubro de 2008, do Decreto nº 6.899, de 15 de julho de 2009, e com as normas editadas pelo Conselho Nacional de Controle de Experimentação Animal (CONCEA), e foi **Aprovado** pela Comissão de Ética no Uso de Animais (CEUA/UFPI) da Universidade Federal do Piauí, em Reunião na presente data **13/10/2022**.

Finalidade	**( ) Ensino (X) Pesquisa Científica**
Vigência da Autorização	**02/01/2023 a 30/12/2027**
Espécie/Linhagem/raça	**-Camundongo heterogênico/Swiss**<br>**-Camundongo isogênico/ Balb/c**
Nº de Animais	**- 1476**<br>**- 808**
Peso/ Idade	**-2 meses/25 – 35 g**<br>**-2 meses/25 – 35 g**
Sexo	**Fêmeas**
Origem	**Biotério Central da UFPI**
Local de alojamento dos animais durante o experimento	**BIOTÉRIO SETORIAL I**
Grau de Invasividade	**2**

Teresina, 16 de Novembro de 2022.

Veruska Cavalcanti Barros

Profa. Dra.Veruska Cavalcanti Barros

Coordenadora da Comissão de Ética no Uso de Animais - CMPP/UFPI
